# An Arginine Finger Regulates the Sequential Action of Asymmetrical Hexameric ATPase in the Double-Stranded DNA Translocation Motor

**DOI:** 10.1128/MCB.00142-16

**Published:** 2016-09-12

**Authors:** Zhengyi Zhao, Gian Marco De-Donatis, Chad Schwartz, Huaming Fang, Jingyuan Li, Peixuan Guo

**Affiliations:** a Division of Pharmaceutics and Pharmaceutical Chemistry, College of Pharmacy, and Department of Physiology and Cell Biology, College of Medicine, The Ohio State University, Columbus, Ohio, USA; b College of Pharmacy, University of Kentucky, Lexington, Kentucky, USA; c Institute of High Energy Physics, Chinese Academy of Sciences, Beijing, China

## Abstract

Biological motors are ubiquitous in living systems. Currently, how the motor components coordinate the unidirectional motion is elusive in most cases. Here, we report that the sequential action of the ATPase ring in the DNA packaging motor of bacteriophage ϕ29 is regulated by an arginine finger that extends from one ATPase subunit to the adjacent unit to promote noncovalent dimer formation. Mutation of the arginine finger resulted in the interruption of ATPase oligomerization, ATP binding/hydrolysis, and DNA translocation. Dimer formation reappeared when arginine mutants were mixed with other ATPase subunits that can offer the arginine to promote their interaction. Ultracentrifugation and virion assembly assays indicated that the ATPase was presenting as monomers and dimer mixtures. The isolated dimer alone was inactive in DNA translocation, but the addition of monomer could restore the activity, suggesting that the hexameric ATPase ring contained both dimer and monomers. Moreover, ATP binding or hydrolysis resulted in conformation and entropy changes of the ATPase with high or low DNA affinity. Taking these observations together, we concluded that the arginine finger regulates sequential action of the motor ATPase subunit by promoting the formation of the dimer inside the hexamer. The finding of asymmetrical hexameric organization is supported by structural evidence of many other ATPase systems showing the presence of one noncovalent dimer and four monomer subunits. All of these provide clues for why the asymmetrical hexameric ATPase gp16 of ϕ29 was previously reported as a pentameric configuration by cryo-electron microscopy (cryo-EM) since the contact by the arginine finger renders two adjacent ATPase subunits closer than other subunits. Thus, the asymmetrical hexamer would appear as a pentamer by cryo-EM, a technology that acquires the average of many images.

## INTRODUCTION

The ASCE (additional strand catalytic E) superfamily, including the AAA+ (ATPases associated with various cellular activities) superfamily, is a broad class of proteins among which are several nano-biological molecular motors, or nanomotors. Nanomotors facilitate a wide range of functions (1–5), many of which are involved in DNA replication, repair, recombination, chromosome segregation, protein degradation, membrane fusion, microtubule severing, peroxisome biogenesis, gene regulation, DNA/RNA transportation, bacterial division, and many other processes (6–10).

Despite their functional diversity, ring-shaped P-loop NTPases share two conserved modules with Walker A and Walker B motifs (11), exerting their activity through the ATP-dependent remodeling for translocation of macromolecules. The Walker A motif is responsible for ATP binding, while the Walker B is responsible for ATP hydrolysis (12, 13). This energy transition can result in either a gain or loss of substrate affinity, therefore generating a mechanical force exerted on the substrate to produce a mechanical motion. This motion will lead to a contact with or a separation from the substrate molecule, resulting in molecule folding/unfolding, complex assembly/disassembly, or translocation of DNA, RNA, protein, or other substrates (2–4, 14).

Both the revolving mechanism and the sequential reaction mechanism adapted by biological systems through evolution are efficient methods of unidirectional translocation of lengthy double-stranded DNA (dsDNA) genomes, with minimum consumption of energy and without tangling or coiling (15–19). However, both the revolving mechanism and/or the sequential reaction mechanism for DNA translocation require signal communication from one component to another in the motor complex. It has been reported that ASCE ATPases contain one arginine finger motif along with the Walker A and Walker B motifs (20–30). In the active ATPase ring, the arginine residue is located in proximity to the gamma-phosphate of the bound ATP in the adjacent ATPase subunit (22, 25–27). An arginine finger has been confirmed to associate with the formation of the ATP binding pocket (24, 27–30). To understand how the motor component coordinates its motion necessary for unidirectional DNA translocation activity and sequential action of the ATPase ring, we analyzed the role of the arginine finger motif in the ATPase core of the dsDNA translocation motor. It was found that this motif controls the formation of the coordinating dimer inside the hexamer of the motor ATPase. The dimer, however, is not static but shifts and alters with time in a sequential manner, and this sequential reaction mechanism is regulated by the arginine finger.

## MATERIALS AND METHODS

### Cloning, mutagenesis, and protein purification.

The engineering of enhanced green fluorescent protein (eGFP)-gp16 and the purification of the gp16 fusion protein have been reported previously (31). Construction of eGFP-gp16 mutants, including arginine finger mutant R146A, Walker A mutant G27D, and Walker B mutant E119A as well as mCherry-gp16 mutant R146A was accomplished by introducing mutations in the gp16 gene by Keyclone Technologies.

### Glycerol gradient ultracentrifugation.

Fifty microliters of eGFP-gp16 (500 μg/ml) was dropped on the top of 5 ml of linear 15 to 35% glycerol gradients in TMS buffer (50 mM Tris-HCl, pH 8.0, 100 mM NaCl, 10 mM MgCl_2_). After centrifugation at 35,000 rpm in an SW55 rotor at 4°C for 22 h, the gradients were collected into 31 fractions from bottom to top and measured using a plate reader under 488-nm excitation before being applied to an *in vitro* assembly assay.

### EMSA.

A fluorescently tagged protein that facilitates detection and purification was shown to possess assembly and packaging activities similar to those of the wild type (31, 32). The electrophoretic mobility shift assay (EMSA) has been described previously (16, 17). The gp16 mutants or the wild type were mixed with 33 bp of Cy5-dsDNA in the presence or absence of ATP and γ-S-ATP. Samples were incubated at ambient temperature for 20 min and then loaded onto a 1% agarose gel (44.5 mM Tris, 44.5 mM boric acid) and electrophoresed at 4°C for around 1 h at 8 V/cm. The eGFP-gp16, mCherry-gp16, and Cy5-DNA samples were analyzed by a fluorescent LightTools whole-body imager using 488-nm, 540-nm, and 635-nm excitation wavelengths for GFP, mCherry, and Cy5, respectively.

### Protein structure prediction and analysis.

I-TASSER (33) was used to predict the structure of the subunit of gp16 through a threading algorithm. The structure prediction processed without restraint, allowing the server to select the template. The N domain (amino acids [aa]1 to 180) of the predicted structure adopts a RecA-like fold, which is the conserved structure for many oligomeric ATPases, including T7 gp4 and FtsK. The root mean square deviation (RMSD) between the predicted structure (N domain of gp16) and FtsK (beta domain) after the structure alignment is around 3 Å. The predicted structure (monomer) was used to construct a hexameric structure of gp16 with Pseudomonas aeruginosa FtsK (Protein Data Bank [PDB] accession number 2IUU) as the template (34). VMD was used to render the image of the structure (35).

### Proteinase probing assay.

Three microliters of His-gp16 (2 mg/ml) was mixed with trypsin (0.5 μg) and different amounts of ATP (0 nmol, 16 nmol, 32 nmol, 64 nmol, 128 nmol, 256 nmol, 512 nmol, and 1 μmol) in the enzyme reaction buffer (50 mM NaCl, 25 mM Tris, pH 8, 0.01% Tween 20, 0.1 mM MgCl_2_, 2% glycerol, 1.5% polyethylene glycol [PEG] 8000, 0.5% acetone, and 5 mM dithiothreitol [DTT]). Fresh DTT was added to the buffer right before the reaction. The final volume for this reaction system was 30 μl; the samples were incubated at room temperature for 30 min and applied on 12% SDS-PAGE gels.

### Intrinsic tryptophan fluorescence assay.

Eight microliters of SUMO-gp16 (1 μg/μl) was incubated with different amounts of ATP in the reaction buffer (0.005% Tween 20, 1.5% PEG 8000, 0.5% acetone, and 2 mM Tris, pH 8.0). The fluorescence intensity of the samples was immediately measured through a spectrofluorometer at a wavelength excitation of 280 nm.

### ATPase activity assay.

Enzymatic activity via fluorescent labeling was described previously (36). Briefly, a phosphate binding protein conjugated to a fluorescent probe that senses the binding of phosphate was used to assay ATP hydrolysis.

### *In vitro* assembly inhibition assay.

Purified *in vitro* components were mixed and were subjected to a virion assembly assay as previously described (37). Briefly, newly assembled infectious virions were inoculated to Bacillus bacteria and plated. Activity is expressed as the number of plaques formed per volume of sample (PFU/milliliter).

## RESULTS

### Hypothesis of motor motion mechanism to be tested.

Most biological motor ATPases assemble into hexameric rings with a motion process stimulated by ATP (11). For the ϕ29 dsDNA translocation motor, our hypothesis is the following: (i) an arginine finger is present in the ϕ29 motor ATPase gp16; (ii) the arginine finger extends to the upstream adjacent ATPase subunit to serve as a bridge for the formation of a dimeric subcomplex and regulates the sequential action of the subunits in the hexameric ATPase ring; (iii) one ATPase dimer and four monomers are present in the hexameric ring; (iv) ATP binding results in the reshaping of the conformation and change of the entropic landscape of gp16; and (v) due to DNA-dependent ATPase activity (11), binding of DNA to the ATP/gp16 complex resulted in ATP hydrolysis, leading to a second conformational change and further entropy alternation of the ATPase to a low-DNA-affinity configuration that allows the release of dsDNA for its concomitant transfer to the adjacent subunit.

**FIG 1:**
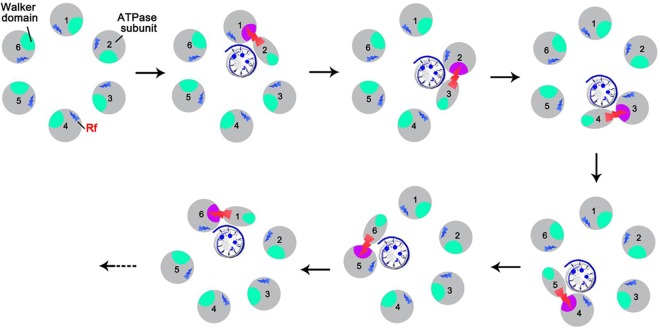
The proposed mechanism of ATPase coordination with a series of conformational changes during DNA binding and ATP hydrolysis that are regulated by the arginine finger (Rf, red).

The model assumes that ATPase undergoes a series of conformational changes during ATP/DNA binding and ATP hydrolysis that are organized in a sequential manner and that this sequential mechanism is coordinated by the arginine finger (Fig. 1), in accordance with the supporting data described below.

### Identification of arginine finger motifs in ϕ29 gp16 ATPase.

**FIG 2:**
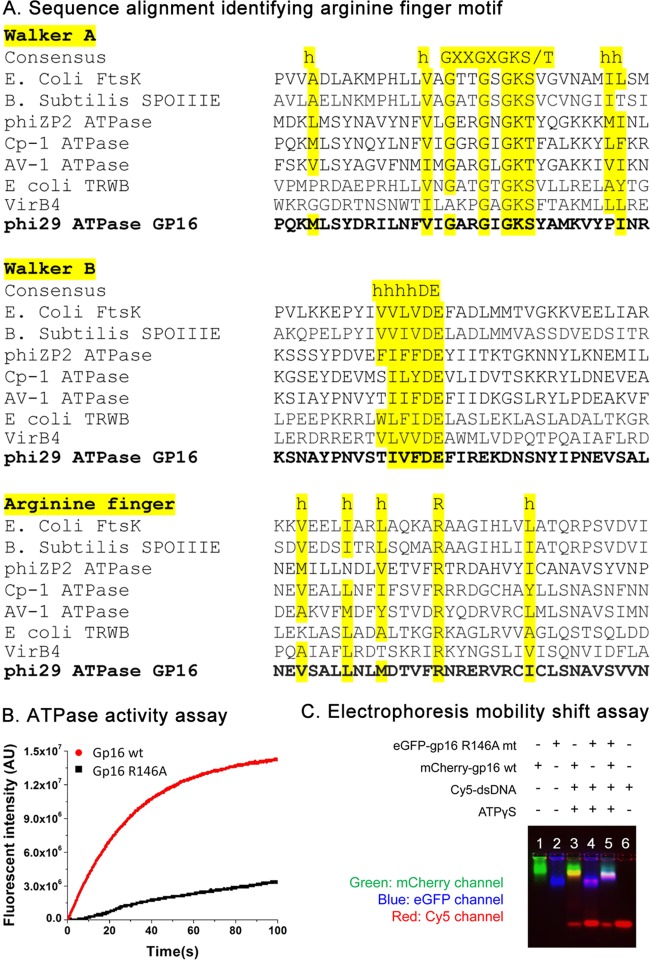
Identification and characterization of the arginine finger in the ϕ29 gp16 ATPase. (A) Sequence alignment among gp16 ATPase and other ATPases in the same family, indicating the location of the Walker A, Walker B, and arginine finger (R) motifs of gp16 ATPase, which are well aligned with previously established domains (11, 27, 34, 39–41). h, hydrophobic residue. (B and C) ATP binding and hydrolysis activity assay of the gp16 arginine mutant. After the R146 residue is mutated, gp16 ATPase loses its ATP hydrolysis activity (B) and DNA binding activity, as shown by EMSA (C). wt, wild type.

gp16 shares the common ATP binding domain typical of all ASCE family members, including AAA+ proteins (2, 38). This domain contains very well conserved motifs responsible for ATP binding and ATP hydrolysis (12), which have been previously identified as Walker A (11) and Walker B motifs (17), respectively. However, detailed information about the arginine finger motif of ϕ29 has remained elusive. Sequence alignment was subsequently performed with similar ASCE family proteins to identify this motif (Fig. 2A). From the alignment, we identified the position of the arginine fingers (residue 146) localized after the position of the beta-4 strand, as seen in other ATPases, which correlates well with the known structural information and consensus sequences for this motif found in other proteins (27, 34, 39–41) (Fig. 2A). The single mutant R146A gp16 was produced and examined for its ATPase activity. As expected, the arginine finger mutant was severely impaired in both ATP hydrolysis activity (Fig. 2B) and DNA binding in the presence of γ-S-ATP (Fig. 2C), possibly due to the impaired affinity for γ-S-ATP, which is similar to that of the Walker A mutant (17). In contrast, the Walker B mutants retained their binding affinity for DNA in the presence of γ-S-ATP and were also sufficient in binding DNA in the presence of ATP, although they could not hydrolyze ATP (16, 17).

### The arginine finger extends to the upstream adjacent ATPase subunit to serve as a bridge for the formation of a dimeric subcomplex and regulates the sequential action of the subunits in the hexameric ATPase ring.

**FIG 3:**
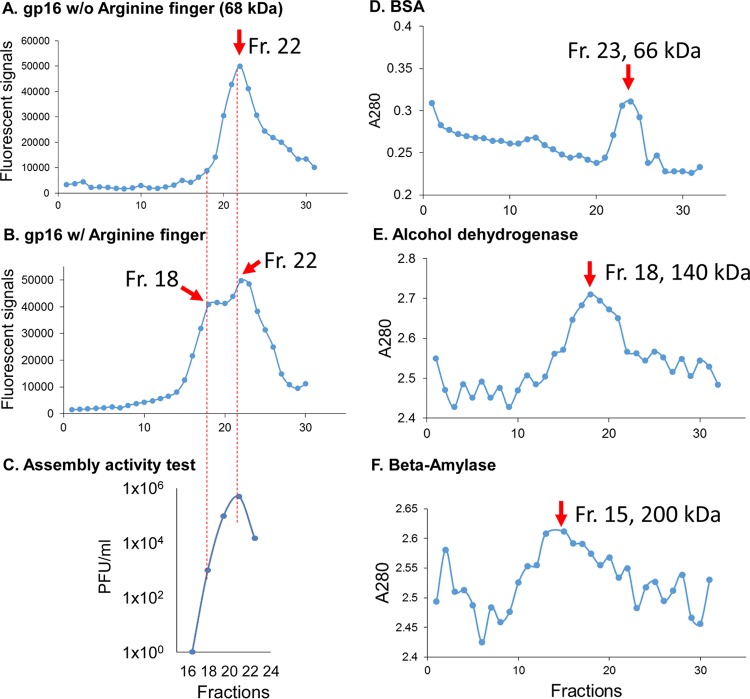
Ultracentrifugation assay showing the presence of both dimers and monomers in gp16 ATPase rings. (A and B) One peak of eGFP-gp16 R146A (A) and two peaks of eGFP-gp16 wild type (B) were shown after parallel ultracentrifugation in a 15% to 35% glycerol gradient, indicating that both monomers and dimers were formed in the gp16 wild type, while dimer formation is interrupted by the mutation of the arginine finger. (C) The isolated gp16 dimers did not show any viral assembly activity, supporting the previous finding that addition of fresh gp16 monomers is required for reinitiating the DNA packaging intermediates. (D to F) Ultracentrifugation fractions (Fr) of protein markers, including BSA (66 kDa), alcohol dehydrogenase (140 kDa), and beta-amylase (200 kDa), are shown, with their peak locations around fractions 23, 18, and 15, respectively, to mark the separation of the monomer and dimer of gp16 ATPase. w/, with; w/o, without.

**FIG 4:**
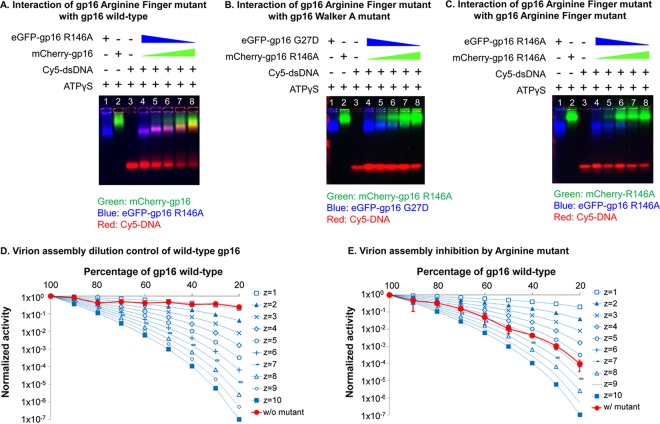
Intersubunit interaction of gp16 arginine mutant with other gp16s. (A to C) EMSAs showing the interaction of the gp16 arginine finger mutant with wild-type gp16 (A), the gp16 Walker A mutant (B), and the arginine finger mutant (C). Interactions between the gp16 arginine finger mutant and wild-type gp16 or the gp16 Walker A mutant are demonstrated by the band shift of both ATPase and DNA in the gel, while no obvious band shifts were observed when the arginine finger mutant ATPases were mixed together. DNA was labeled with Cy5, and different ATPases were labeled with different fluorescent protein tags for observation in the gel. (D and E) Binomial distribution assay to show the blockage of the ATPase arginine finger mutant on motor packaging activity. Different ratios of buffer (D) or eGFP-gp16 arginine finger mutants (E) were mixed with wild-type gp16 ATPase for the *in vitro* virion assembly activity assay. The experimental curve is plotted with theoretical predictions made according to the equation of Fang et al. (59) The experimental curve matches with the theoretical prediction with *z* = 6, indicating that six subunits are present in the ATPase ring, and one arginine finger mutant is enough to block the activity of the motor (see Materials and Methods).

The arginine finger has been reported to have various functions, including a major role in subunit communications by pivoting upon ATP hydrolysis to trigger the conformational changes of the subunits of ATPase (23, 42–46). The formation of the dimeric complex of gp16 in the absence of ATP was demonstrated by different approaches: glycerol gradient ultracentrifugation (Fig. 3), electrophoretic mobility shift assay (EMSA) (Fig. 4A to C), size exclusion chromatography, and native gel electrophoresis (17). These assays were based on the previous finding that fusion of the GFP protein to the N terminus of gp16 did not interfere with activity of the ATPase gp16 in DNA packaging (31, 47, 48). It was found that mutation of the arginine finger abolished dimer formation within the ATPase (Fig. 3). Although the arginine mutants alone could not form dimers, interactions were observed when the arginine mutants were mixed with either the wild type or mutants that contained an intact arginine finger, which can provide an arginine residue for dimer formation (Fig. 3). The disruptive effect of the arginine finger mutation on assembly ability was also reflected in protein activity since it was observed that one single inactive subunit of an arginine finger mutant was able to completely inactivate the whole ATPase ring in an assembly inhibition assay (Fig. 4D and E). This supports the idea that in the ATPase ring, one adjacent wild-type ATPase provided an arginine finger to interact with the arginine mutant and that the lack of one arginine in the entire ring completely abolished the activity of the whole ring.

**FIG 5:**
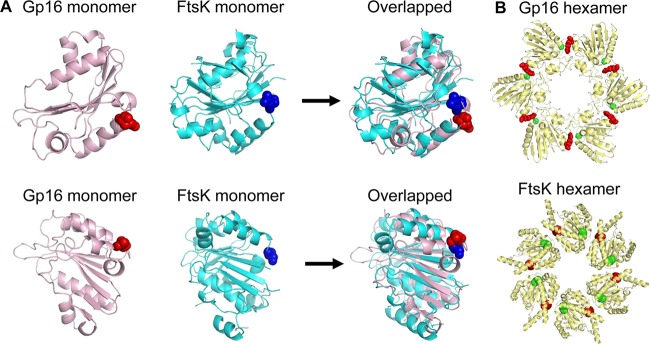
Prediction and comparison of gp16 structure. (A) Structural comparison between the crystal structure of FtsK monomer (PDB accession number 2IUU; cyan) and the gp16 ATPase model (pink). The arginine finger is highlighted as a sphere. (B) Comparison of the predicted gp16 hexamer and FtsK hexamer. The ATPase gp16 hexamer structure was constructed using the predicted monomer structure shown in panel A and the P. aeruginosa FtsK (PDB accession number 2IUU) as templates (34). VMD was used to render the image of the structure (35). The ATP domains are highlighted as spheres: residue 27 (green, the conserved Walker ATP domain) and residue 146 (red, the arginine finger). The interaction of the arginine finger with the upstream adjacent subunit is evidenced by the proximity of the red and green spheres in both the constructed structure of the gp16 hexamer and the FtsK hexamer crystal structure.

To get a better understanding of the structural role of the arginine finger, we modeled a gp16 hexameric ring using I-TASSER (33) and Phyre2 software (81). The gp16 sequence aligned well with the crystal structure of the hexameric FtsK DNA translocase of Escherichia coli (Fig. 5). Using this model, we observed that the position of the arginine finger of one subunit of gp16 extends to the active site of a neighboring subunit. The predicted structure showed that the arginine finger was part of the ATP binding pocket (Fig. 5). The structural model provides an explanation for the observed cooperative behavior in the hexameric ring of gp16. Not surprisingly given the importance in the formation of the active site, mutations in arginine fingers greatly impaired the ability of gp16 to bind to ATP, to hydrolyze ATP (Fig. 2B), to bind to DNA (Fig. 2C), and consequently to package the DNA (Fig. 4E).

### Both dimer and monomer forms were present in gp16 hexamer.

As demonstrated in the above sections, the arginine finger serves as a bridge between two independent subunits, thus forming a transient dimeric subunit. In wild-type gp16, it was observed that both dimer and monomer forms were present in solution, as revealed by glycerol gradient centrifugation experiments. The molecular masses relative to such fractions were confirmed by protein marker calibrations in the same assay (the 66-kDa bovine serum albumin [BSA] localized around fraction 23, the 140-kDa alcohol dehydrogenase localized around fraction 18, and the 200-kDa beta-amylase localized at around fraction 15). We thus proceeded to test the packaging activity of the different fractions of the gp16 ATPase recovered from the gradient. Interestingly, it has been observed that DNA packaging activity was retained with the fractions containing monomers, while fractions containing only dimers displayed no DNA packaging activity (Fig. 3C). These results agree with the finding that the addition of fresh gp16 monomer to the DNA packaging intermediates is required for reinitiating motor DNA packaging activity and the conversion of the intermediates into infectious viruses (49).

### ATP binding resulted in the change of conformation and entropic landscape of gp16.

**FIG 6:**
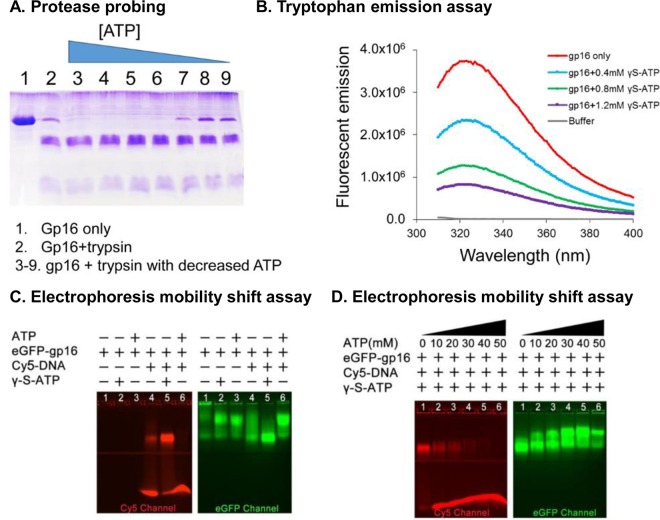
Demonstration of two separate steps of gp16 conformational changes and entropic landscape alteration after ATP binding and ATP hydrolysis, respectively. (A) Trypsin probing showed that the ATPase-digested band is decreased with a reduced amount of ATP added into gp16 ATPase samples, suggesting that the gp16 ATPase is less constrained after binding to ATP. (B) Intrinsic tryptophan fluorescence assay showing the signal changes of ATPase upon the addition of different concentrations of ATP. (C) EMSA showing that gp16 ATPase bound to ATP and undergoes a conformational change that has a high affinity for DNA and that ATP hydrolysis triggers a second conformational change of gp16 ATPase with a low affinity with DNA. (D) Increasing DNA is released from gp16 ATPase/DNA/ATP complex upon the addition of increased amount of ATP that can be hydrolyzed by the gp16 ATPase.

ASCE family proteins undergo a cycle of conformational changes during ATP binding and hydrolysis with basically two major states: high or low affinity for the DNA substrate. In recent publications (16, 17, 32, 50), we proposed a similar model for gp16, in which binding to ATP exerted an effect on the conformational state of the protein that predisposed it to binding to DNA (high affinity). Conversely, ADP would promote another conformational state in which DNA binding is not favorable. This notion, together with the observation that the arginine finger has a role in regulating both the conformational state of gp16 and its interaction with the adjacent subunit, prompted us to question whether the effect of ATP binding on gp16 was able to modify not only the conformation of the DNA binding portion of the protein but also the structural characteristics of gp16 altogether. We thus tested if ATP binding was able to alter the shape of gp16 by partial proteolysis treatment and by intrinsic tryptophan fluorescence assay (Fig. 6A and B). Interestingly, both assays indicated a conformational change in the gp16-ATP complex. Moreover, as visible from the partial proteolysis test, protection from proteolysis is indicative of a larger population of gp16 with a constrained conformation before ATP binding.

An electrophoretic mobility shift assay was also employed to study the interaction between ATPase and dsDNA in the presence of γ-S-ATP, a nonhydrolysable ATP analog. Stronger binding of gp16 to dsDNA was observed when gp16 was incubated with γ-S-ATP (Fig. 6C), suggesting that the gp16/dsDNA complex is stabilized through addition of the nonhydrolysable ATP substrate.

### Hydrolysis of ATP transformed the ATPase into a second conformation with low affinity for dsDNA, thus pushing the dsDNA toward an adjacent ATPase subunit.

Consequent to the first structural change, it was also observed that the binding of the ATP/gp16 complex to DNA resulted in ATP hydrolysis and also the passage to a second conformational change with a low-DNA-affinity configuration (11, 16, 36, 51). This explains the finding in 1987 that the ϕ29 DNA packaging protein gp16 is a DNA-dependent ATPase (11). Such a state resulted in the release of dsDNA for its concomitant transfer to the adjacent subunit. The conclusion was also supported by the finding that the addition of normal ATP promoted the release of dsDNA from the gp16–γ-S-ATP–dsDNA complex (Fig. 6D).

## DISCUSSION

ϕ29 genomic DNA packaging involves multiple components, including a 12-subunit connector, a hexameric prohead RNA (pRNA) ring (52, 53), and an ASCE ATPase gp16 hexamer. Great interest has arisen about this packaging system for its intriguing mechanism of action and for its useful applications in nanotechnology (54–60). It has been demonstrated that pRNA works as a point of connection between ATPase and the connector (61) and that the hexameric ATPase (16, 17) provides the pushing force for the packaging of genomic DNA, acting in coordination with the connector that acts as a one-way valve (50, 62, 63).

Nanobiomotors have been previously classified into two main categories: linear and rotational motors. These two categories have been clearly documented in single-molecule imaging and X-ray crystallography (64–69). Recently, it has been discovered that the ϕ29 dsDNA packaging motor uses a revolving mechanism that does not require rotation or coiling of the dsDNA (15–17, 70). The discovery of a revolving mechanism establishes a third class of biomotors. This finding resolves many puzzles and debates that have arisen throughout the history of painstaking studies on the motor (19, 70).

The ATPase hexameric ring exerts a force, pushing the dsDNA in a sequential manner to advance through the dodecamer channel, which acts as a one-way valve as reported for the phi29 motor (5, 19, 62, 70, 71). The interest in the sequential revolving mechanism lies in the fact that it elegantly integrates all the known functional and structural information about the packaging core (the ATPase, pRNA, and connector). Moreover, it offers solutions for many questions that arise from investigations of the DNA packaging phenomenon (i.e., coordination between energy consumption and DNA packaging and the ability to translocate a long strain of dsDNA without coiling or tangling). However, in order to have a sequential mechanism (which has been proposed for many proteins belonging to the AAA+/ASCE family) (30, 72, 73), several conditions need to be fulfilled. The most important are the following: (i) only one or two subunits of the oligomer are able to bind the substrate with the same affinity exhibited in the entire hexamer; (ii) both the ATPase activity and translocation activity need to demonstrate negative cooperativity when one subunit is able to bind ATP and is not able to hydrolyze the nucleotide (as in the case of the Walker B mutation); (iii) only the ATP-bound state of the protein is the unique state that efficiently binds to DNA.

**FIG 7:**
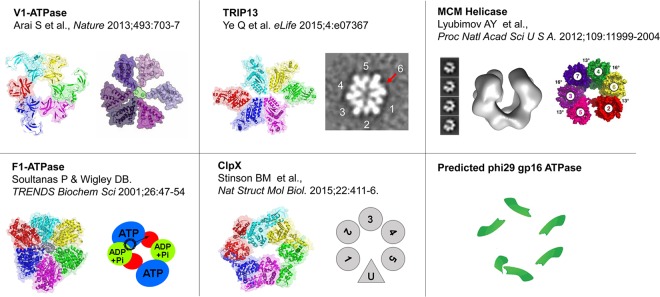
Asymmetrical structure of various ATPase hexamer models. Structure illustrations of V1-ATPase (adapted from reference 75 with permission of the publisher), TRIP13 (adapted and modified from reference 76 with permission of the publisher), ClpX (adapted and modified from reference 77 with permission of the publisher), MCM helicase (adapted from reference 79 with permission of the publisher), and F1-ATPase (80) are shown as representatives of asymmetrical hexamers. PDB accession numbers are as follows: V1-ATPase, 3VR5; TRIP13, 4XGU; F1-ATPase, 1BMF; ClpX, 4I81. The EM reconstruction of the MCM helicase is deposited in the EMDataBank under accession number EMD-5429.

We demonstrated that, indeed, this is the case for the ϕ29 motor ATPase (16, 32). One important question that then arises with the demonstration of the sequential mechanism is how the different subunits of the ATPase can sense the ATP binding/DNA binding state of others. In the present work, we addressed this question by identifying the arginine finger motifs of the ATPase gp16 by sequence alignment and proved that the arginine finger is an essential motif that participates in the formation of the ATP binding pocket by examining the behavior of gp16 mutants with the arginine finger removed. The gp16 mutated in the arginine finger was unable to package DNA, to hydrolyze ATP, or to bind to DNA. The profile of gp16 in ultracentrifugation indicated the presence of a mixture of monomeric and dimeric forms. Mutation of the arginine finger eliminated the capacity of gp16 to assemble into dimeric forms. Arginine finger motifs were thus shown to link two subunits to each other since the arginine motif of one subunit participates in the formation of the ATP binding site of the next subunit (Fig. 7). The importance of the dimer, moreover, is evident, as shown by the DNA packaging assay, in which a reconstituted hexamer of gp16 can efficiently pack DNA inside the procapsid only when ultracentrifuged fractions containing both dimeric and monomeric gp16 are mixed together (data not shown) (49).

In the sequential action of gp16, we proposed that one subunit of the hexamer binds to the DNA, subsequently hydrolyzing ATP to perform a translocation of a certain number of base pairs of DNA (11, 74). The DNA is then passed to the subsequent subunit, and the process is repeated. It is intriguing to notice that the position and function of ATPase offer the possibility of carrying the information of ATP/DNA binding from one ATPase subunit to another, with the cooperative behavior of gp16 seen in the case of other mutants (Walker B mutations) (16).

The sequential action mechanism of the ϕ29 ATPase is essential for optimal translocation efficiency. This mechanism integrates well with our overall model of the revolving motor and a “push through one-way valve” model (16, 50). Without coordination during the energy production of gp16, the cycles of binding and release of DNA would create futile cycles of ATP hydrolysis, inhibiting the unidirectional translocation process (15, 16, 32). Arginine fingers thus act as integrators of information for the entire process of DNA packaging. Years of evolution have created an efficient biomotor, one that can be used in the future for applications in nanotechnology (54–60).

Furthermore, the conclusion that coordination is provided by an asymmetrical hexamer was supported by structural computation, X-ray diffraction, and cryo-electron microscopy (cryo-EM) imaging of other hexameric ATPase systems (Fig. 7) (71, 75–80). These results could provide some clues as to why the asymmetrical hexameric ATPase of gp16 of ϕ29 and gp17 of T4 was previously interpreted as a pentameric configuration by cryo-EM. Since the two adjacent subunits of the ATPase could interact with each other and form a closer dimer configuration, this dimer will appear as a monomeric subunit different from the others, and the hexameric ring will be asymmetrical (Fig. 7).
